# Determinants of demand for intelligent ankle-foot orthoses in children with cerebral palsy: a multicenter cross-sectional study from a multi-stakeholder perspective

**DOI:** 10.3389/fpubh.2026.1855559

**Published:** 2026-06-09

**Authors:** YongPing Yao

**Affiliations:** 1 Tianjin University, Tianjin, China; 2 Sichuan Nursing Vocational College, Chengdu, China

**Keywords:** assistive technology, cerebral palsy, demand analysis, intelligent ankle-foot orthosis, multicenter study, public health

## Abstract

**Background:**

Intelligent ankle-foot orthoses (AFOs) have emerged as a promising assistive technology for improving mobility in children with cerebral palsy (CP). However, their effective development and implementation require a comprehensive understanding of user needs from multiple stakeholders, particularly in resource-limited regions.

**Objective:**

To systematically investigate the demand characteristics for intelligent AFOs among families of children with CP and medical professionals in Sichuan Province, and to identify key determinants influencing these demands.

**Methods:**

A multicenter cross-sectional survey was conducted, including 95 families of children with CP and 39 medical professionals. Structured questionnaires were used to assess functional demands, usage experiences, and perceived barriers. Data were collected from multiple hospitals and rehabilitation centers across different administrative regions in Sichuan Province to enhance representativeness. Group differences were analyzed using non-parametric tests, and multivariate logistic regression was applied to identify independent determinants of demand.

**Results:**

Families demonstrated the highest demand for practical and usability-oriented functions, particularly walking stability (93.69%) and ease of donning and doffing (83.16%). In contrast, medical professionals prioritized clinically oriented functions, including gait monitoring and predictive fall prevention (both 87.18%). Significant inter-group differences were observed in data monitoring (*p* < 0.001) and adaptive adjustment (*p* = 0.012). Multivariate analysis revealed that higher household income was significantly associated with increased demand for both data monitoring functions (OR = 3.13, 95% CI: 1.15–8.58, *p* = 0.026) and usability-related features (OR = 3.00, *p* = 0.033).

**Conclusion:**

Demand for intelligent AFOs exhibits clear stakeholder-specific patterns, reflecting a divergence between practicality-driven needs among families and clinically oriented expectations among medical professionals. Safety, comfort, and indoor scenario adaptability represent shared priorities, while economic constraints and prior technology exposure further shape demand. These findings highlight the necessity of integrating multi-stakeholder perspectives into device design and support the development of context-specific strategies to improve accessibility and adoption in resource-limited settings.

## 1 Introduction

Cerebral palsy (CP) is a common neurodevelopmental disorder in childhood. Most affected children have varying degrees of motor dysfunction, among which gait abnormalities caused by ankle joint disorders (e.g., foot drop, equinovarus) are the most common (1). Ankle-foot orthoses (AFOs), as important assistive devices for improving gait function in children with CP, can stabilize the ankle joint and correct abnormal posture, thereby enhancing walking ability and independence in daily living (2).

However, traditional AFOs are mostly based on a rigid fixation principle. Although they have some effect in correcting abnormal posture, they have obvious limitations in dynamically adapting to gait changes. At the same time, they are associated with problems such as poor wearing comfort, restricted mobility, and decreased long-term adherence (3). With the development of wearable sensing technology and artificial intelligence algorithms, intelligent AFOs are emerging. By integrating functions such as gait monitoring, dynamic adjustment, and fall prediction, they can better adapt to the child’s motor status, providing a new technological pathway for CP rehabilitation (4).

Although intelligent AFOs have promising application prospects, their development and dissemination must be based on a thorough understanding of user needs. Existing studies have mostly focused on the biomechanical performance or clinical efficacy of the devices, with insufficient attention given to the differences in demand among different stakeholders (e.g., families of affected children and medical professionals) and the factors influencing those differences. Moreover, regional disparities in economic development and healthcare resource distribution may further affect the acceptance and functional preferences for intelligent AFOs.

Sichuan Province, as a populous region in western China, has a large number of children with CP. Studies have shown that the overall incidence of CP among children in China is approximately 2.3‰ (4), while a survey in the Aba region of Sichuan reported 2.7‰ (5), indicating a high potential demand in this area. In addition, relatively low household income levels and uneven distribution of medical resources in the region may significantly influence the functional requirements and price acceptability of intelligent AFOs.

Regarding technology acceptance, relevant research indicates that the adoption of new medical technologies depends not only on their clinical effectiveness but also on multiple factors such as ease of use, economic burden, and the user’s level of awareness. Therefore, a systematic analysis of demand characteristics from a multi-stakeholder perspective, and further identification of the key determinants, is of great importance for guiding the locally adapted development and precise dissemination of intelligent AFOs.

Based on this, the present study was conducted in multiple medical and rehabilitation institutions in Sichuan Province. Using a multicenter cross-sectional questionnaire survey, we systematically analysed the demand characteristics for intelligent AFOs among families of children with CP and medical professionals, and further explored the influencing factors, in order to provide evidence-based support for product design optimization and regional promotion strategies of intelligent AFOs.

## 2 Materials and methods

### 2.1 Study design

This study employed a multicenter cross-sectional questionnaire design. Data were collected from participating healthcare and rehabilitation institutions in Sichuan Province, China, including tertiary hospitals and primary rehabilitation centers, to ensure regional representativeness and sample heterogeneity.

### 2.2 Participants

#### 2.2.1 Children with cerebral palsy and their families

Inclusion criteria: (1) children diagnosed with cerebral palsy (CP) according to established criteria (6); (2) aged 1–18 years; (3) residing in Sichuan Province; (4) currently or previously using an ankle-foot orthosis (AFO); and (5) whose guardians provided informed consent.

Exclusion criteria: (1) children with severe cognitive impairment or uncontrolled epilepsy preventing AFO use; and (2) families unable to complete the questionnaire due to special circumstances (e.g., relocation or family disruption).

A total of 95 children with CP were included, and 95 valid questionnaires were collected (response rate: 100%). No sex-based quota sampling was applied. The approximately balanced sex distribution resulted from consecutive recruitment of eligible children who met the inclusion criteria during the data collection period.

#### 2.2.2 Medical professionals

Inclusion criteria: (1) ≥ 1 year of clinical experience in rehabilitation-related departments in Sichuan Province; (2) professional background in rehabilitation medicine, physical therapy, occupational therapy, or orthotics; and (3) familiarity with AFO-related functions.

Exclusion criteria: (1) individuals engaged only in administrative or research work without direct clinical experience with CP patients; and (2) those unable to complete the questionnaire during the study period.

A total of 39 medical professionals were included, with a response rate of 100%. A total of 39 medical professionals were included, with a response rate of 100%. The sample size of medical professionals was determined by the availability of eligible participants across the participating institutions during the study period, rather than by a formal *a priori* power calculation.

### 2.3 Survey instruments

Two structured questionnaires were developed based on previously published assistive technology needs assessment tools (7, 8) and adapted to the regional context of Sichuan Province. In this study, intelligent AFOs were defined as ankle-foot orthoses that retain the basic supportive and corrective functions of conventional AFOs while integrating intelligent functions such as sensor-based gait monitoring, adaptive adjustment, gait-related feedback, and fall-risk warning or prediction. This definition was used consistently in both the family questionnaire and the medical professional questionnaire.

The family questionnaire consisted of six sections (33 items): (1) demographic and clinical characteristics (e.g., age, GMFCS level, AFO usage, household income, and living environment); (2) functional demand assessment using a 5-point Likert scale (1 = not important at all, 5 = very important), including domains such as walking stability, adaptive adjustment, and data monitoring; (3) daily living needs and difficulties with conventional AFO use; (4) expectations and acceptance (e.g., acceptable price range and warranty preferences); (5) open-ended questions; and (6) willingness to participate in trials and provide feedback.

The medical professional questionnaire included five sections: (1) demographic and professional characteristics (e.g., age, specialty, years of experience, and AFO prescription experience); (2) functional demand assessment using a 5-point Likert scale; (3) importance of clinical evaluation indicators (e.g., walking speed and fall risk); (4) perceived technical barriers and improvement priorities; and (5) preferred application scenarios and operational modes (Table 1).

**Table 1:** Comparison of core functional demand for intelligent AFOs between families of children with cerebral palsy and medical professionals.

Functional domain	Group	*n*	High demand (%) (Likert 4–5)	Mean ± SD	*U* value	*p*-value
Walking stability	Families	95	93.69	4.68 ± 0.59	1,523	0.287
	Medical professionals	39	87.18	4.51 ± 0.72		
Ease of donning and doffing	Families	95	83.16	4.42 ± 0.81	1,234	0.043
	Medical professionals	39	38.46	3.85 ± 1.12		
Data monitoring	Families	95	60.00	3.45 ± 1.24	856	<0.001
	Medical professionals	39	87.18	4.49 ± 0.68		
Adaptive adjustment	Families	95	80.00	4.35 ± 0.89	945	0.012
	Medical professionals	39	60.00	3.72 ± 1.15		
Comfort optimization	Families	95	83.16	4.38 ± 0.91	1,289	0.065
	Medical professionals	39	87.18	4.44 ± 0.76		

*p* < 0.05, *p* < 0.01.

Scores were measured on a 5-point Likert scale (1 = not important at all, 5 = very important). *p*-values were obtained using the Mann–Whitney *U* test.

The reliability and validity of both questionnaires met the requirements for medical survey instruments, as confirmed by Cronbach’s *α* coefficient and KMO and Bartlett’s tests (9).

### 2.4 Data collection and quality control

Data collection was conducted from August 2025 to September 2025, using a standardized multicenter approach across participating institutions in Sichuan Province. All investigators received unified training on questionnaire administration, communication techniques, ethical considerations, and data quality control procedures.

GMFCS level was collected based on caregiver report. When caregivers were unable to provide this information, the response was recorded as “unknown”; no additional clinician-confirmed GMFCS assessment was performed before questionnaire completion.

Eligible participants were informed of the study objectives, and written informed consent was obtained prior to participation. Before completing the questionnaire, all participants received a standardized briefing on the meaning of intelligent AFOs. The briefing included a written definition and a neutral verbal explanation by trained investigators. Intelligent AFOs were introduced as AFO devices incorporating functions such as gait monitoring, adaptive adjustment, comfort optimization, and fall-risk warning or prediction. All participants received the same explanation, and investigators were instructed not to provide individualized examples or persuasive explanations that could influence participants’ responses. No visual aids, device prototypes, or live demonstrations were used during the briefing. For respondents with limited literacy, trained investigators provided neutral explanations to ensure comprehension while avoiding response bias.

Completed questionnaires were checked on-site for completeness and logical consistency. Incomplete or inconsistent responses were corrected or excluded to ensure data quality.

### 2.5 Variable definitions

The dependent variable was the functional demand for intelligent AFOs, assessed using a 5-point Likert scale. Scores ≥4 were defined as “high demand.”

Independent variables included demographic and clinical characteristics. For families, variables included household income, awareness of the Gross Motor Function Classification System (GMFCS), and AFO usage experience. For medical professionals, variables included years of experience, professional background, experience with dynamic AFOs, and patient volume (Table 2).

**Table 2:** Comparison of priority of clinical evaluation indicators between families and medical professionals.

Evaluation indicator	Priority level (families)	Priority level (medical professionals)	Interpretation
Fall risk assessment	High (93.69%)	High (87.18%)	Strong agreement
Patient satisfaction	High	High	Strong agreement
Walking speed improvement	Moderate	High	Greater emphasis by medical professionals on quantitative outcomes
Plantar pressure distribution	Low	High	Clinically oriented focus among medical professionals
Social function improvement	Low	High	Greater attention to quality-of-life outcomes among medical professionals

Differences between groups were assessed using the Mann–Whitney *U* test. No significant difference was observed for fall risk assessment (*p* > 0.05), while significant differences were found for walking speed improvement and plantar pressure distribution (*p* < 0.05).

### 2.6 Statistical analysis

Statistical analyses were performed using SPSS version 26.0. Categorical variables were presented as frequencies and percentages, while continuous variables were expressed as mean ± standard deviation. Internal consistency was assessed using Cronbach’s *α* coefficient, and construct validity was evaluated using the Kaiser–Meyer–Olkin (KMO) test and Bartlett’s test of sphericity.

Group comparisons were conducted using the Mann–Whitney *U* test or Kruskal–Wallis *H* test, depending on data distribution. Given the heterogeneity between families and medical professionals, these comparisons were considered exploratory.

Variables with *p* < 0.10 in univariate analysis were included in multivariate logistic regression models to identify independent determinants of demand. Separate models were constructed for families and medical professionals. No formal *a priori* power analysis was conducted before data collection. Therefore, the sample size should be interpreted as a pragmatic sample based on available eligible participants in the participating institutions. Given the relatively small sample size of medical professionals (*n* = 39), a simplified logistic regression model was applied to reduce model complexity and avoid overfitting. Results from this model were considered exploratory. Results were reported as odds ratios (ORs) with 95% confidence intervals (CIs) (Table 3).

**Table 3:** Multivariate logistic regression analysis of determinants of demand for intelligent AFOs among families of children with cerebral palsy.

Variable	OR	95%CI	*p*-value
Household income	3.13	1.15–8.58	0.026
GMFCS awareness	5.55	0.61–50.74	0.129
Dissatisfaction with conventional AFOs	0.56	0.14–2.22	0.411

OR, odds ratio; CI, confidence interval.

A two-sided *p*-value <0.05 was considered statistically significant.

Variables with *p* < 0.10 in univariate analysis were included in multivariate logistic regression models to identify independent determinants of demand. Separate models were constructed for families and medical professionals. No formal *a priori* power analysis was conducted before data collection. Therefore, the sample size should be interpreted as a pragmatic sample based on available eligible participants in the participating institutions.

For the medical professional group, candidate variables initially considered for regression analysis included professional background, years of clinical experience, monthly number of children with CP treated, experience prescribing conventional AFOs, experience with dynamic AFOs, and confidence in prescription decision-making. However, because of the limited sample size of medical professionals and the small number of outcome events, only two clinically relevant variables were retained in the simplified logistic regression model: experience with dynamic AFOs and years of clinical experience. These variables were selected because they were considered directly related to clinicians’ familiarity with AFO technologies and their ability to evaluate the clinical value of intelligent AFO functions. The model was therefore intended to provide exploratory rather than confirmatory evidence. Results were reported as odds ratios (ORs) with 95% confidence intervals (CIs).

### 2.7 Ethics statement

This study was conducted in accordance with the Declaration of Helsinki and was approved under a registered clinical research project entitled “Clinical study on intelligent ankle-foot orthosis based on a cloud platform for the treatment of lower limb motor dysfunction in children with cerebral palsy” (Registration No.: ChiCTR2000032597).

Although the registered project involved multiple pilot institutions, the present study was conducted only in participating institutions within Sichuan Province.

Written informed consent was obtained from all participants or their legal guardians prior to data collection. All data were anonymized to ensure confidentiality.

## 3 Results

### 3.1 Reliability and validity of the questionnaires

The internal consistency of both the family questionnaire and the medical professional questionnaire was assessed using Cronbach’s *α* coefficient, with values of 0.744 and 0.816, respectively (Table 4).

**Table 4:** Multivariate logistic regression analysis of determinants of demand for ease of donning and doffing.

Variable	OR	95%CI	*p-*value
Household income	3.00	1.09–8.25	0.033
GMFCS awareness	—	—	>0.05
Dissatisfaction with conventional AFOs	1.08	0.24–4.90	0.917

For the family questionnaire, the Kaiser–Meyer–Olkin (KMO) value was 0.718, and Bartlett’s test of sphericity yielded an approximate *χ*^2^ value of 161.002 (*p* < 0.001). For the medical professional questionnaire, the KMO value was 0.742, and Bartlett’s test yielded an approximate *χ*^2^ value of 361.591 (*p* < 0.001).

These results indicate that both questionnaires demonstrated acceptable reliability and validity for subsequent analysis.

### 3.2 Results of the family questionnaire

#### 3.2.1 Basic characteristics of children with CP and AFO usage

Among the 95 children included in the study, the gender distribution was balanced, and the majority were aged 0–7 years. Among the 95 children included in the study, the gender distribution was balanced, and the majority were aged 0–7 years. Notably, 64 families (67.37%) were unaware of the child’s Gross Motor Function Classification System (GMFCS) level, indicating a substantial gap in caregiver awareness of standardized functional classification. Most children resided in urban areas, although a substantial proportion lived outside major cities within Sichuan Province.

In terms of family characteristics, primary caregivers were predominantly parents, and most households reported a monthly income of less than 10,000 RMB.

Regarding AFO usage, 67.37% (64/95) of children were currently using AFOs, primarily rigid types. Daily wearing time was generally less than 2 h, and overall satisfaction was reported as moderate. The main issues reported with conventional AFOs included excessive weight and difficulty in donning and doffing. Detailed data are presented in Supplementary Tables 1, 2.

#### 3.2.2 Functional demand for intelligent AFOs

**Figure 1:**
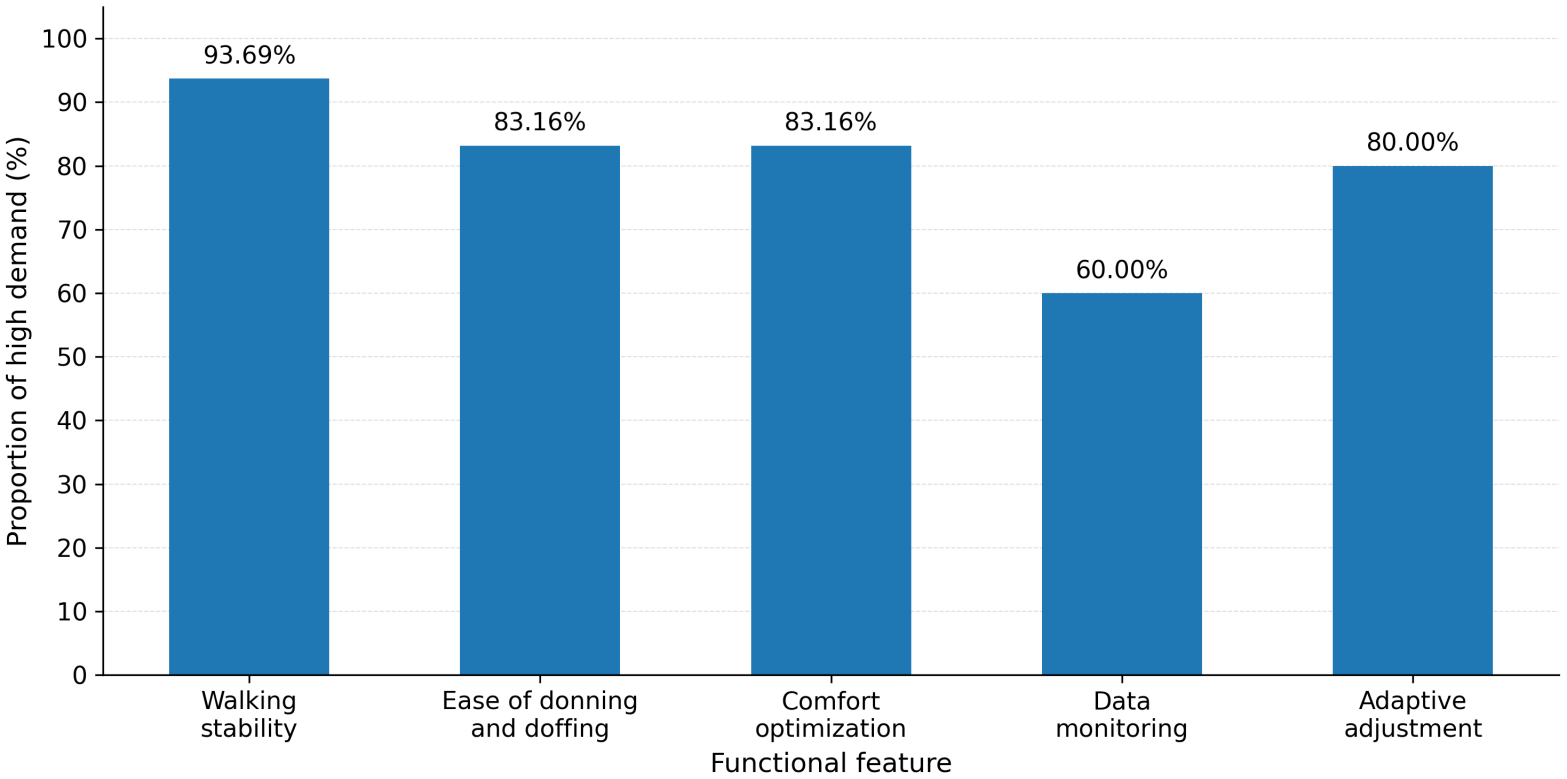
Proportion of high demand (Likert score 4–5) for intelligent AFO functional features among families of children with cerebral palsy.

Among families of children with CP, the highest demand was observed for walking stability, with 93.69% (89/95) reporting high demand (Likert score 4–5). High demand was also observed for ease of donning and doffing and comfort optimization, both at 83.16% (79/95). In addition, 80.00% (76/95) of respondents reported high demand for adaptive adjustment functions, whereas demand for data monitoring was relatively lower at 60.00% (57/95) (Figure 1).

These findings indicate that families prioritize practical features that directly improve usability and daily experience.

**Figure 2:**
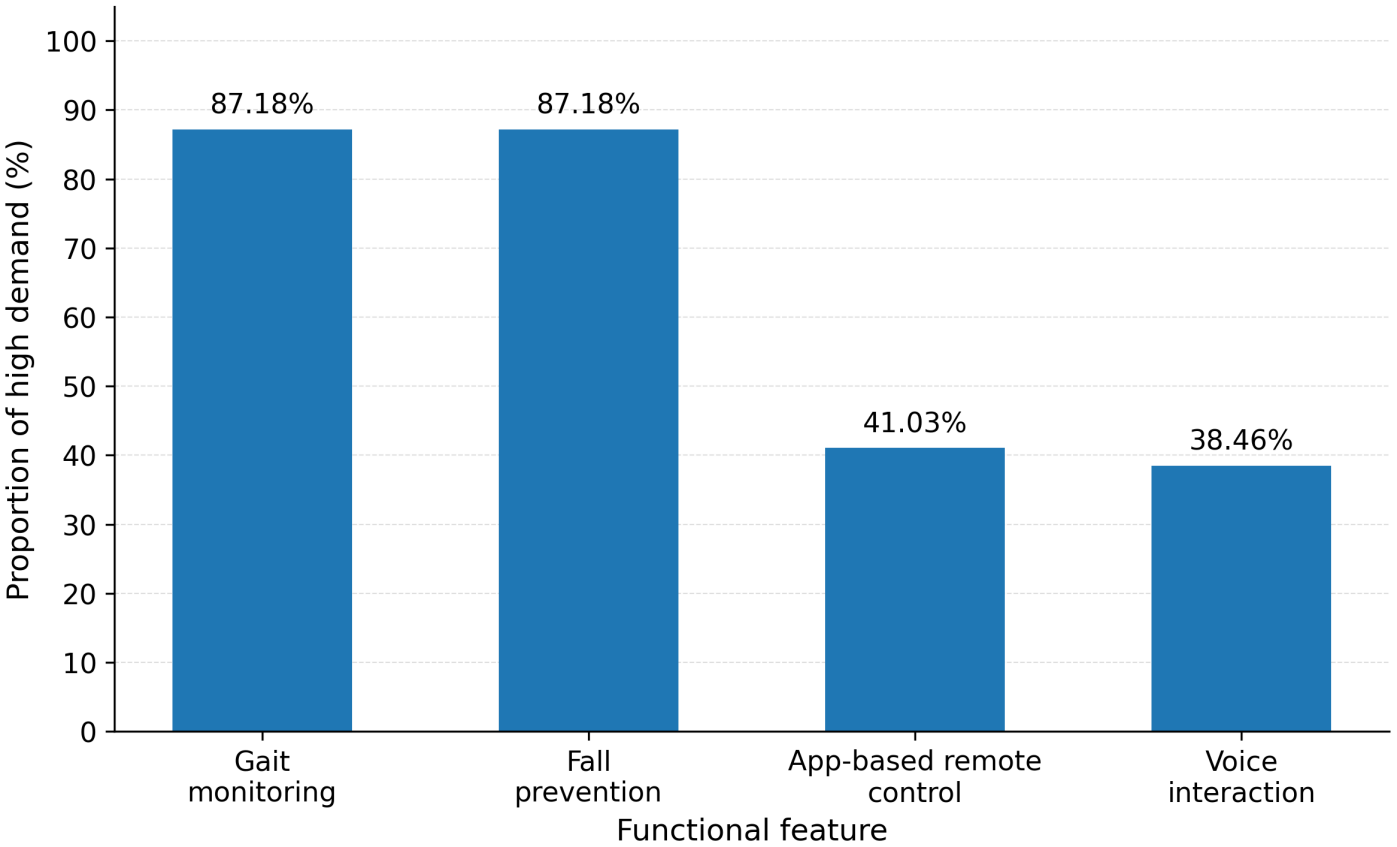
Proportion of high demand (Likert score 4–5) for intelligent AFO functional features among medical professionals.

Among medical professionals, high demand was reported for gait monitoring and predictive fall prevention, both at 87.18% (34/39). In contrast, demand for interactive features such as app-based remote control and voice interaction was lower, at 41.03% (16/39) and 38.46% (15/39), respectively (Figure 2).

These results suggest that medical professionals place greater emphasis on clinically relevant functions, particularly those related to assessment and risk prevention (Table 5).

**Table 5:** Simplified logistic regression analysis of determinants of demand for intelligent AFOs among medical professionals.

Variable	OR	95%CI	*p*-value
Experience with dynamic AFOs	0.15	0.03–0.91	0.039
Years of clinical experience (≥5 years)	3.08	0.58–16.25	0.186

The dependent variable was high demand for real-time gait monitoring and analysis (Likert score 4–5 = 1; 1–3 = 0). Because of the limited sample size, only two clinically relevant independent variables were included to reduce model complexity.

**Figure 3:**
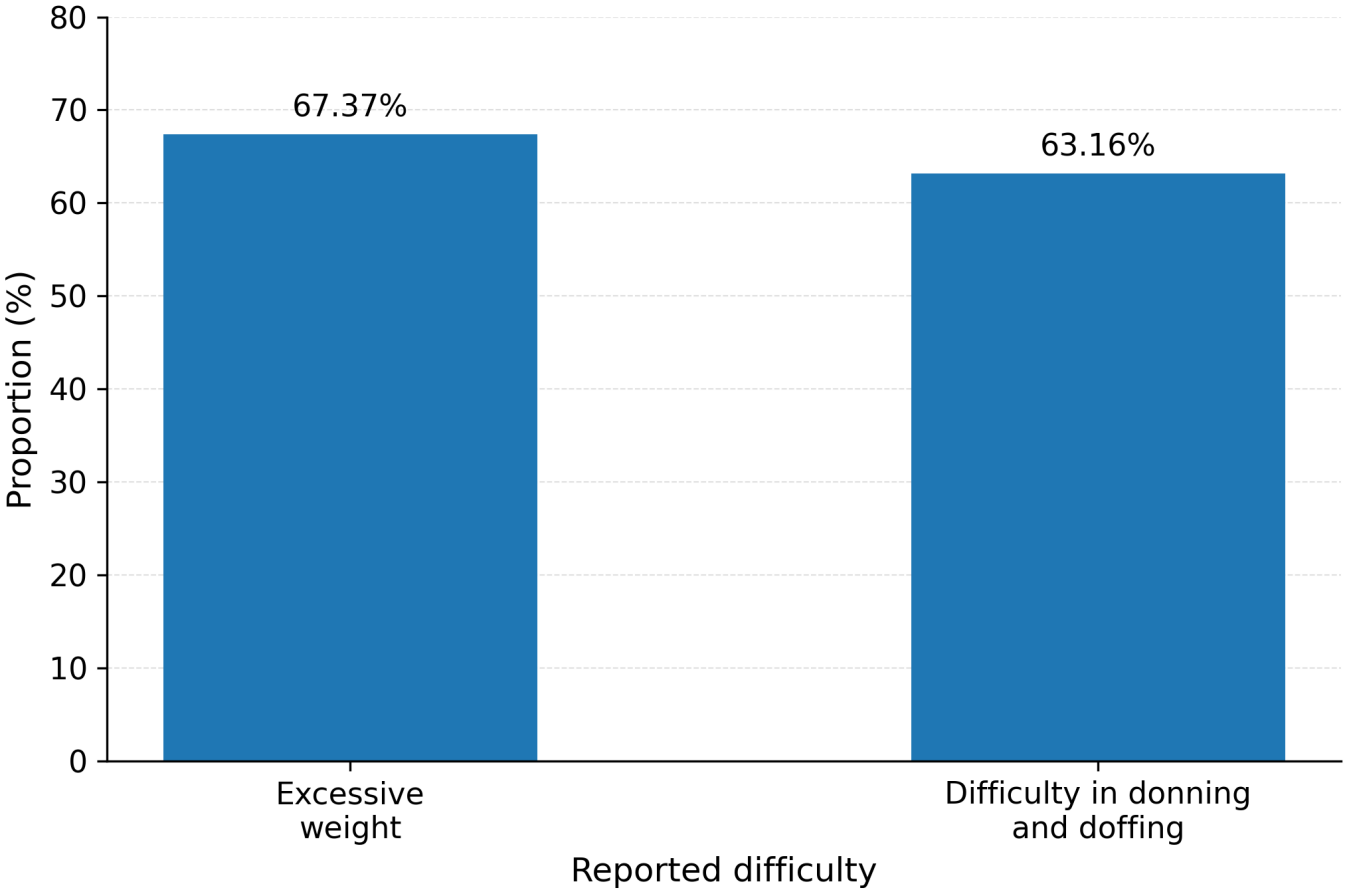
Distribution of reported difficulties in the use of conventional AFOs among families of children with cerebral palsy.

Regarding difficulties with conventional AFOs, “excessive weight” and “difficulty in donning and doffing” were the most frequently reported issues, accounting for 67.37% (64/95) and 63.16% (60/95), respectively. Additional concerns included discomfort and restricted mobility. These findings highlight that limitations in convenience and comfort remain major barriers to long-term adherence (Figure 3).

#### 3.2.3 Willingness to participate and functional demand for intelligent AFOs

Families demonstrated a high level of willingness to participate in the use of intelligent AFOs. The proportion of respondents who reported being “willing” or “very willing” to participate in a free trial of intelligent AFOs reached 80%. In addition, 67.36% (64/95) expressed willingness to provide regular feedback on usage experience. Most families reported either a positive or neutral attitude toward the usability of intelligent AFOs. Regarding warranty expectations, the majority preferred lifetime warranty coverage (Supplementary Table 4).

**Figure 4:**
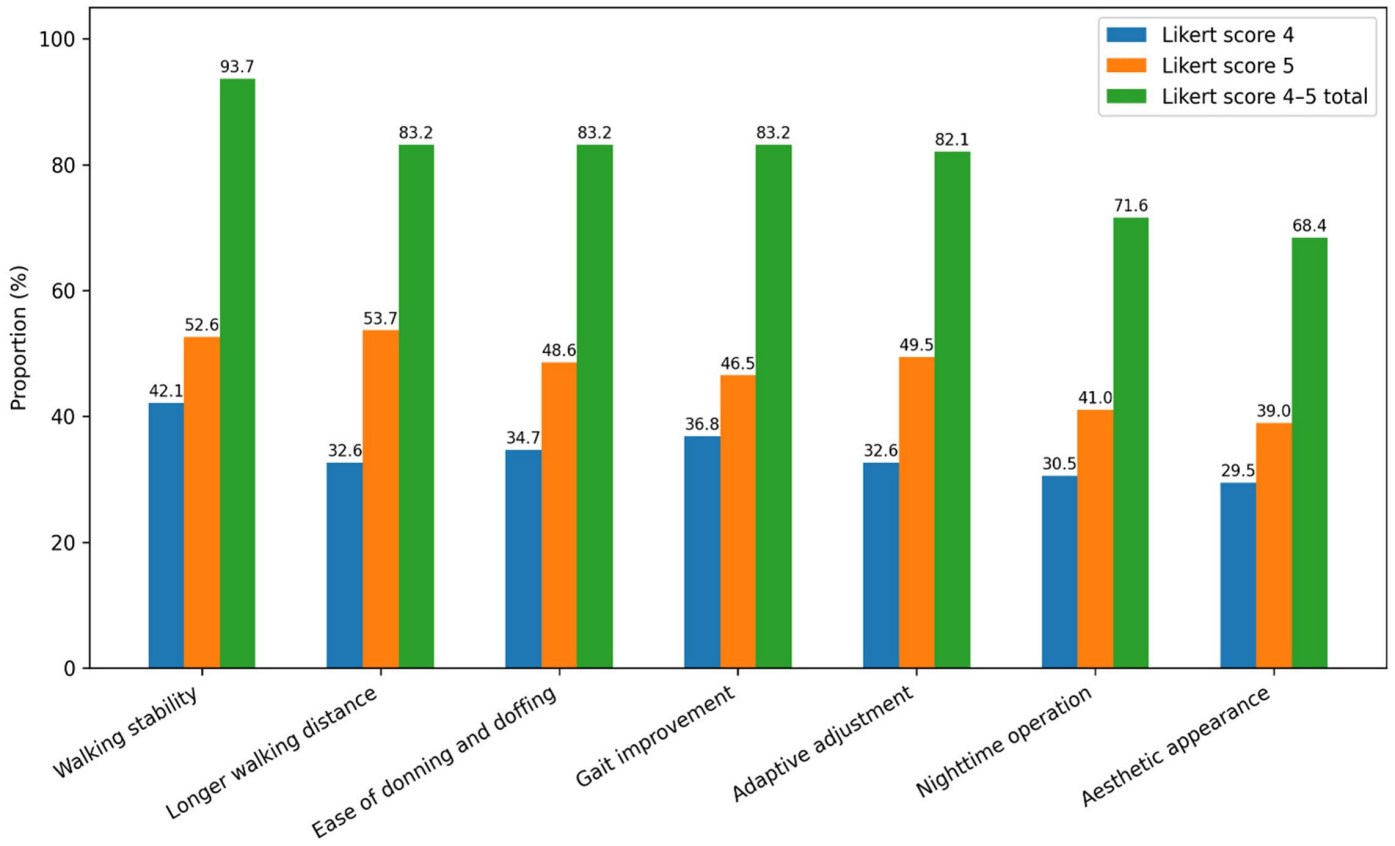
Proportion of high demand (Likert score 4–5) for intelligent AFO functional features among families of children with cerebral palsy.

In terms of functional demand, core needs were primarily concentrated on practicality and convenience. The highest proportion of high demand (Likert score 4–5) was observed for “improving walking stability,” followed by “enabling longer walking distance” and “ease of donning and doffing,” all exceeding 80%. In contrast, demand for “nighttime operation” and “aesthetic appearance” was relatively low (Figure 4).

### 3.3 Results of the medical professional survey

#### 3.3.1 Demographic characteristics and AFO prescription experience

A total of 39 medical professionals were included in the study. In terms of demographic and professional characteristics, females accounted for a higher proportion of the sample. The majority of participants were young to middle-aged (25–44 years), accounting for 94.87, and 58.97% resided in cities outside major urban centers within Sichuan Province.

Physical therapists and rehabilitation physicians constituted the main professional groups, with 66.67% having 1–10 years of work experience, indicating a relatively young workforce with strong professional relevance.

Regarding clinical workload and prescription experience, 76.92% of participants reported treating fewer than 30 children with CP per month. A total of 79.49% (31/39) had experience prescribing conventional AFOs, while 43.59% (17/39) had experience using dynamic AFOs.

In terms of functional demand, medical professionals showed a stronger preference for features related to data monitoring, gait analysis, and clinical evaluation, reflecting a high appreciation for the role of intelligent AFOs in rehabilitation assessment. Confidence in prescription decision-making was predominantly neutral (20/39, 51.28%), indicating an overall cautious attitude (Supplementary Table 5).

#### 3.3.2 Functional demand for intelligent AFOs

The highest-demand functions among medical professionals included personalized gait pattern learning, predictive fall prevention, multi-joint coordinated control, and optimization of wearing comfort, highlighting a strong focus on safety and adaptability.

**Figure 5:**
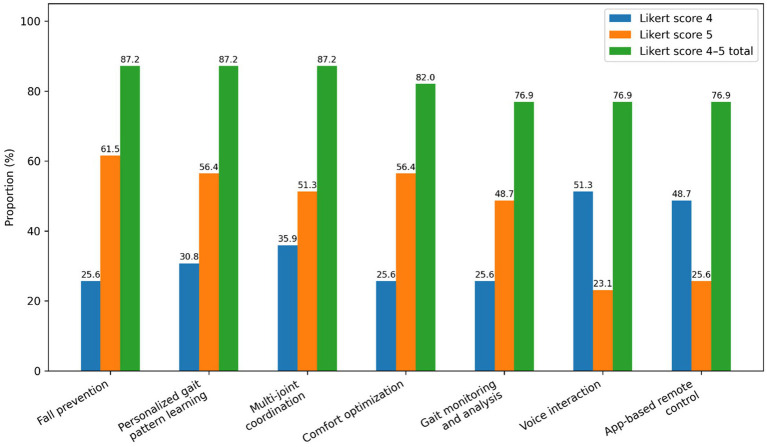
Proportion of high demand (Likert score 4–5) for intelligent AFO functional features among medical professionals.

In contrast, lower demand was observed for features such as voice interaction interfaces and app-based remote control (Figure 5).

#### 3.3.3 Clinical evaluation indicators, technical barriers, and application scenarios

Fall risk assessment and improvement in social function were identified as the primary clinical evaluation indicators, followed by patient satisfaction and plantar pressure distribution, highlighting a strong emphasis on safety and rehabilitation outcomes (Figure 5).

**Figure 6:**
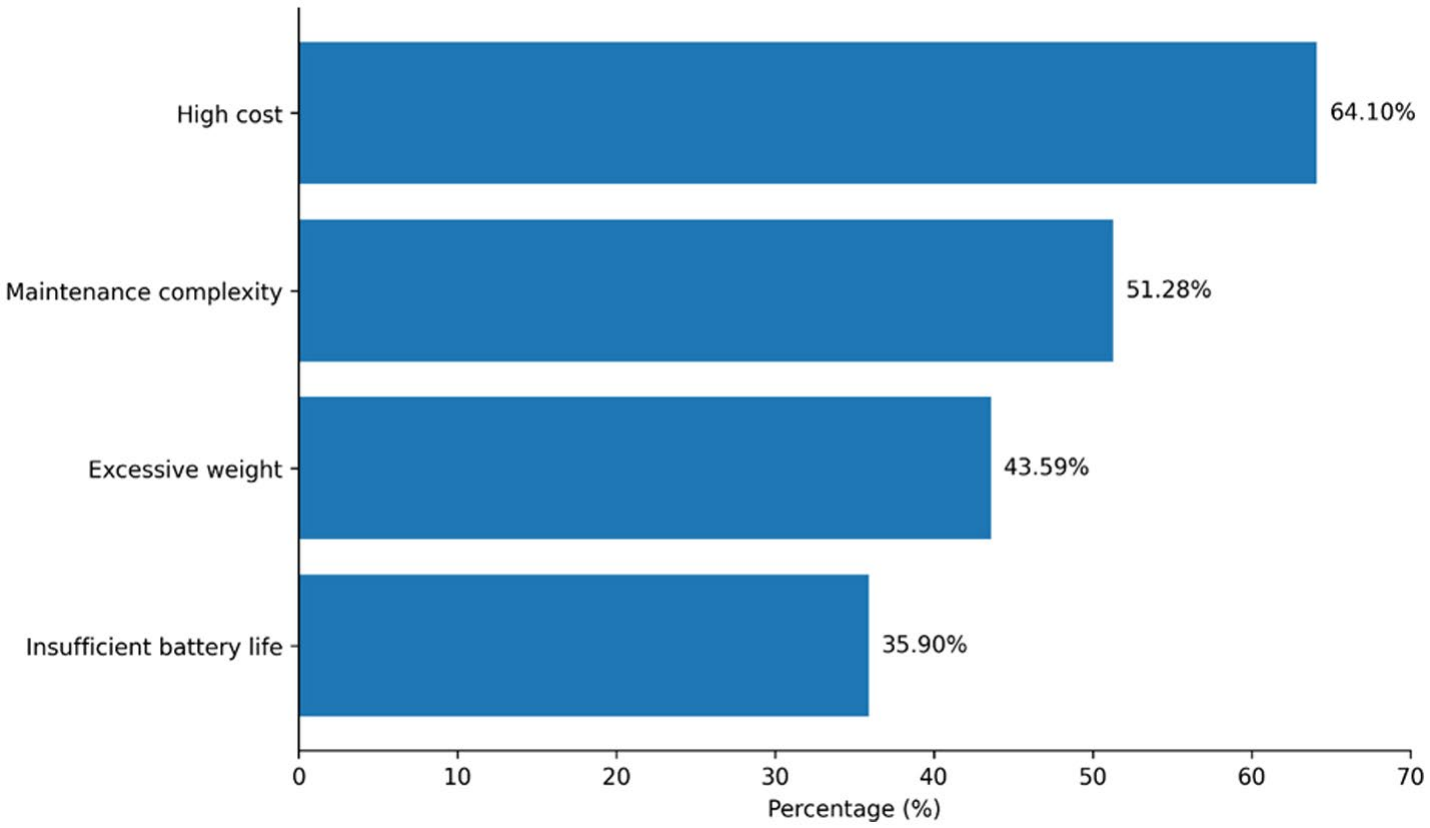
Analysis of technical barriers to intelligent AFOs.

**Figure 7:**
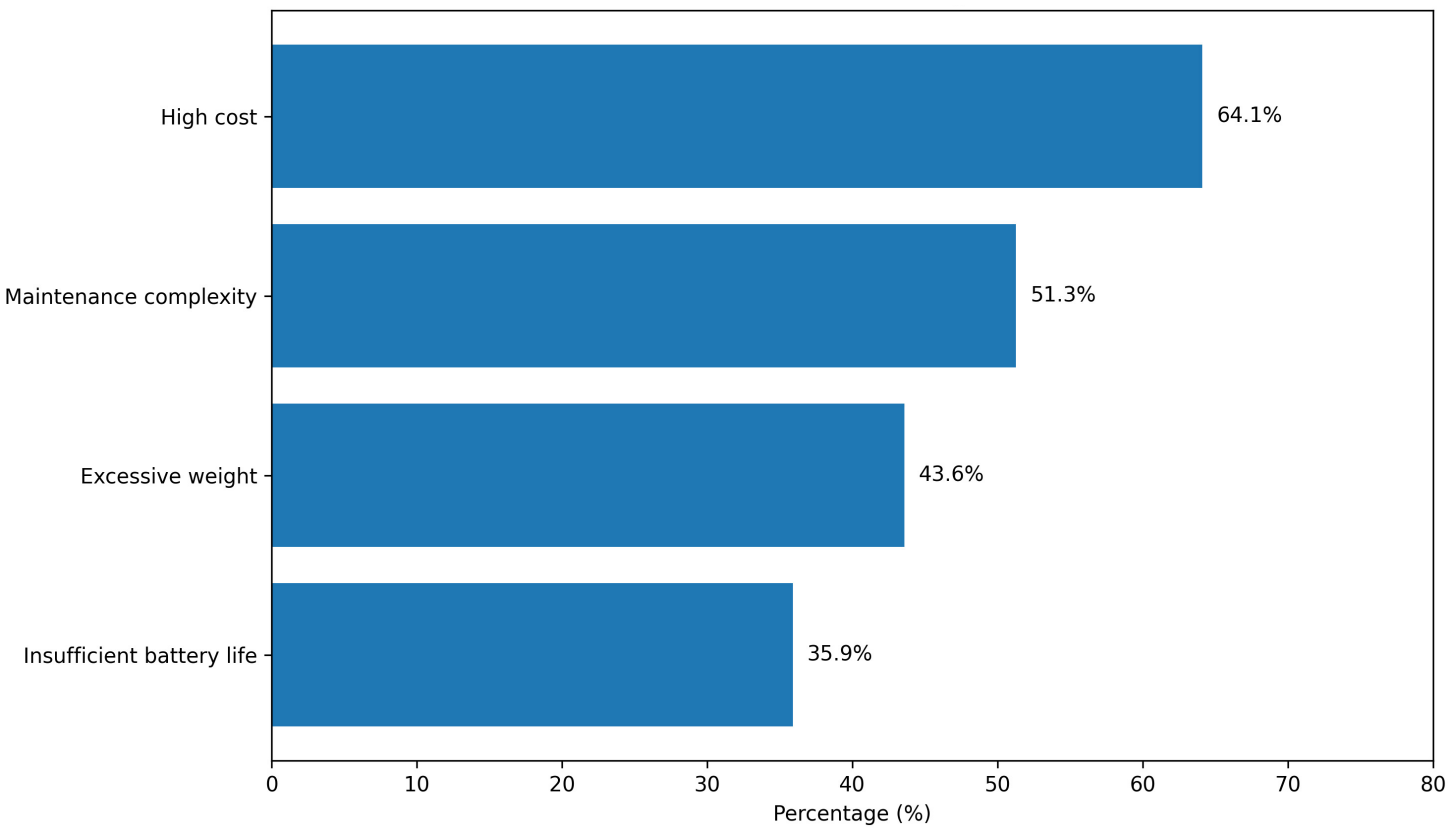
Analysis of technical barriers and expected improvement directions.

In terms of technical barriers (Figure 6) and future development directions (Figure 7), high cost and complex maintenance were identified as the main obstacles. Key areas for technological advancement included the development of lightweight materials and long-lasting battery technologies.

**Figure 8:**
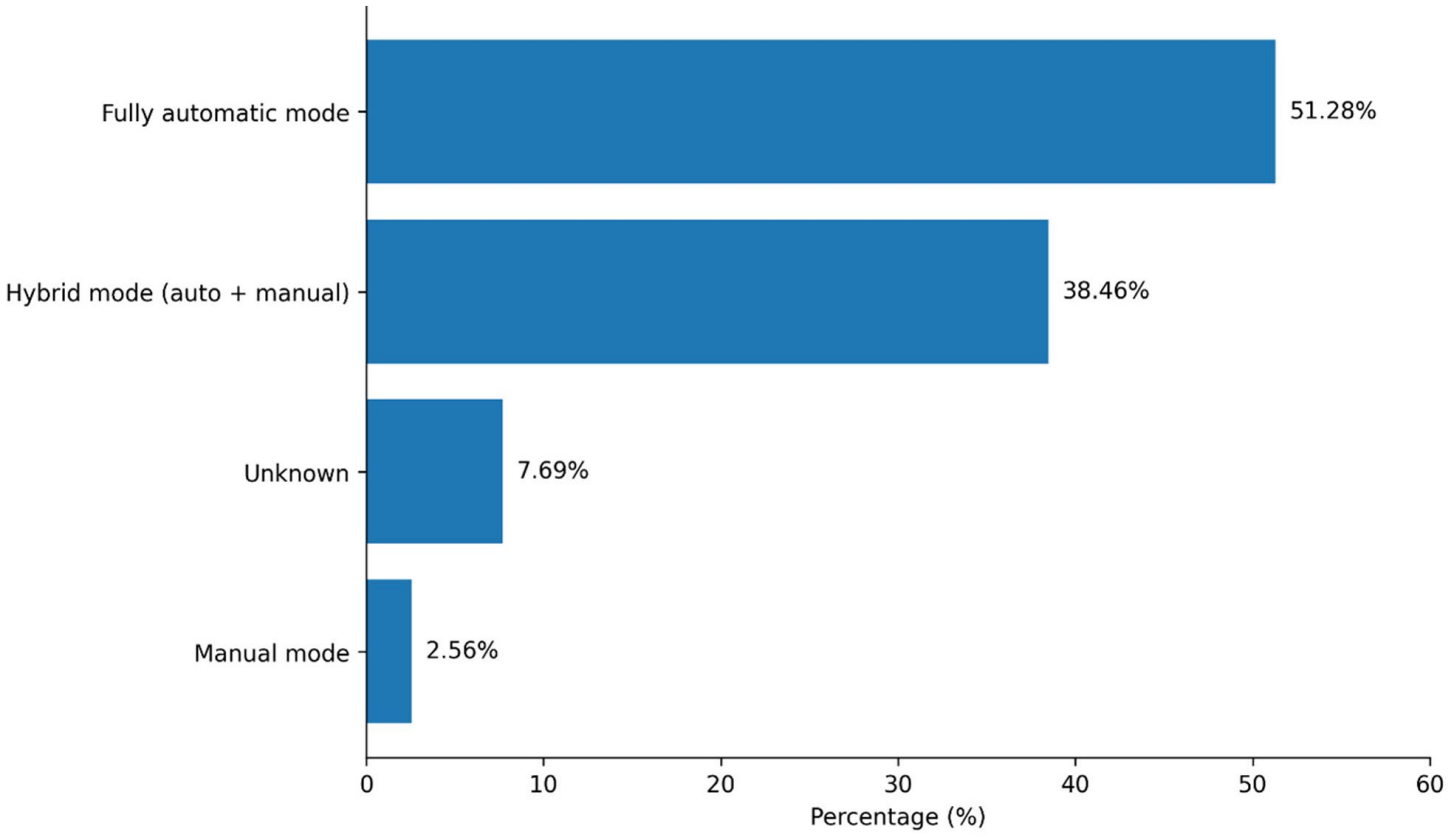
Preferred working modes of intelligent AFOs.

**Figure 9:**
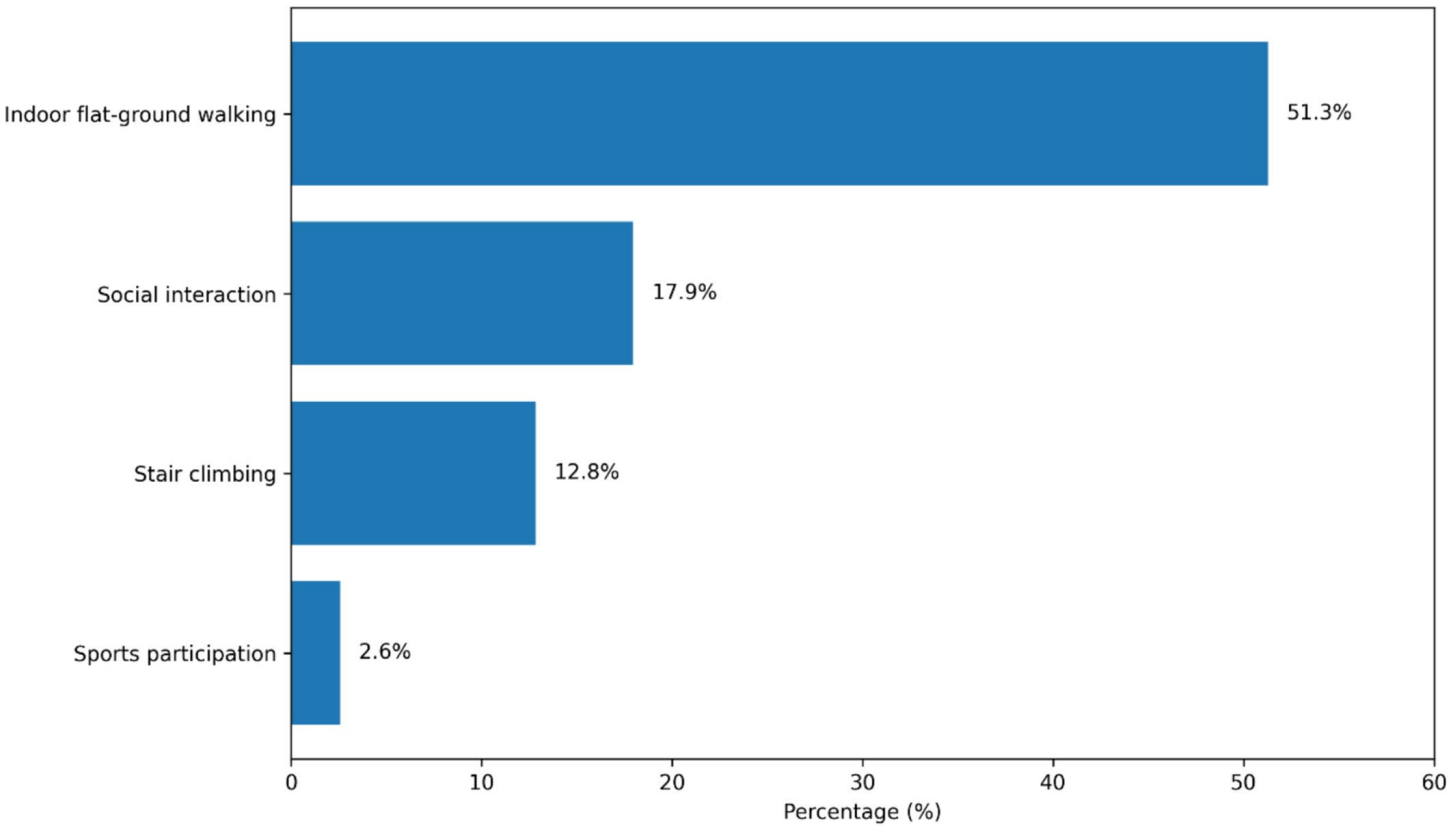
Preferred application scenarios and working modes of intelligent AFOs.

Regarding application scenarios and preferred operational modes of intelligent AFOs, indoor flat-ground walking was identified as the highest-priority scenario, accounting for more than half of responses. In contrast, scenarios such as stair climbing and sports activities accounted for a smaller proportion of responses (Figures 8, 9). Although Sichuan includes many mountainous areas, this result suggests that respondents prioritized the most frequent and basic daily walking scenario rather than more complex terrain-related activities. For preferred operational modes, the fully automatic mode was the most favored, followed by the hybrid mode, while demand for manual mode was minimal.

### 3.4 Exploratory comparison of demand characteristics between stakeholders

To explore differences in demand between families of children with cerebral palsy and medical professionals, the Mann–Whitney *U* test (non-parametric test) was used to compare independent samples across the same functional dimensions.

#### 3.4.1 Comparison of core functional demand between families and medical professionals

In the exploratory analysis, differences in functional demand were observed between families of children with cerebral palsy and medical professionals across multiple dimensions. No significant difference was found in ratings for walking stability (*p* = 0.287), suggesting a shared emphasis on safety.

Families reported significantly higher scores for ease of donning and doffing compared with medical professionals (*p* = 0.043). In contrast, medical professionals assigned significantly higher scores to data monitoring functions (*p* < 0.001).

In addition, families demonstrated higher demand for adaptive adjustment (*p* = 0.012), whereas ratings for comfort optimization were comparable between the two groups (*p* = 0.065).

These findings indicate that different stakeholder groups prioritize distinct functional aspects of intelligent AFOs.

#### 3.4.2 Comparison of clinical evaluation indicators between families and medical professionals

Families and medical professionals showed both shared and distinct priorities regarding clinical evaluation indicators. Both groups placed high priority on fall risk assessment and patient satisfaction, suggesting a common emphasis on safety and user-centered outcomes. However, medical professionals assigned greater importance to quantitative and clinically oriented indicators, including walking speed improvement, plantar pressure distribution, and social function improvement. These differences indicate that families tended to focus more on practical safety and perceived experience, whereas medical professionals placed greater emphasis on measurable rehabilitation outcomes and clinical assessment indicators.

#### 3.4.3 Multivariate logistic regression analysis of determinants of demand for intelligent AFOs

To further identify key factors influencing the demand for intelligent AFOs, a multivariate logistic regression model was constructed based on data from families of children with cerebral palsy.

In the model for demand for ease of donning and doffing, household income was significantly associated with high demand (OR = 3.00, *p* = 0.033), indicating that families with higher income levels placed greater emphasis on device usability.

Similarly, in the model for demand for data monitoring functions, household income remained a significant determinant (OR = 3.13, 95% CI: 1.15–8.58, *p* = 0.026). In contrast, awareness of GMFCS classification and satisfaction with conventional AFOs were not significantly associated with demand (*p* > 0.05).

These findings suggest that economic factors play an important role in shaping demand for intelligent AFO functions.

#### 3.4.4 Simplified logistic regression analysis of demand for intelligent AFOs among medical professionals

To further explore factors influencing demand for intelligent AFOs among medical professionals, an exploratory simplified logistic regression model was constructed due to the limited sample size. The model was intended to provide preliminary evidence rather than confirmatory inference. High demand for “real-time gait monitoring and analysis” (Likert score 4–5) was used as the dependent variable, while “experience with dynamic AFOs” and “years of clinical experience” were included as independent variables.

The results showed that experience with dynamic AFOs was significantly associated with high demand for real-time gait monitoring and analysis (OR = 0.15, 95% CI: 0.03–0.91, *p* = 0.039). In contrast, years of clinical experience were not significantly associated with demand in this model (OR = 3.08, 95% CI: 0.58–16.25, *p* = 0.186).

## 4 Discussion

### 4.1 Shared demand characteristics between families and medical professionals

Based on the Mann–Whitney *U* test, both families and medical professionals demonstrated strong agreement across three key dimensions: safety, comfort, and scenario adaptability. These shared priorities reflect both the clinical characteristics of cerebral palsy and the limitations of conventional AFOs.

(1) Consensus on safety

No significant difference was observed in ratings for walking stability (*p* = 0.287), indicating strong agreement between the two groups. This reflects the increased fall risk in children with cerebral palsy due to impaired balance. Consistent with this, families emphasized walking stability, while medical professionals prioritized fall prevention and risk assessment.

These findings suggest that functions such as real-time gait monitoring and fall prediction are central to intelligent AFO design.

(2) Consensus on comfort

Both groups expressed similar demand for comfort optimization (*p* = 0.065). Previous studies have shown that rigid AFOs may restrict natural ankle motion and reduce walking endurance. In this study, families reported excessive weight and difficulty in donning and doffing as key limitations, while medical professionals also emphasized wearing comfort.

This convergence reflects a shift toward balancing biomechanical correction with user experience.

(3) Consensus on scenario adaptability

Both groups prioritized indoor flat-ground walking despite the mountainous geography of Sichuan. This likely reflects the limited mobility range of children with cerebral palsy, whose daily activities are primarily confined to indoor environments.

Overall, these findings highlight the importance of prioritizing safety, comfort, and real-life usage scenarios in intelligent AFO design.

### 4.2 Differences in demand between families and medical professionals

Despite shared priorities, significant differences were observed between families and medical professionals, reflecting their distinct roles in the rehabilitation process.

(1) Practicality vs. clinical value

Families showed significantly higher demand for usability-related features such as ease of donning and doffing (*p* = 0.043), longer walking distance, and adaptive adjustment. These preferences likely reflect caregiving burden and limited access to professional rehabilitation support.

In contrast, medical professionals placed greater emphasis on data monitoring (*p* < 0.001), personalized gait learning, and multi-joint coordination, reflecting a focus on quantitative assessment and precision rehabilitation.

Experience with dynamic AFOs was significantly associated with demand for gait monitoring functions. This may indicate that clinicians with prior experience have a more critical understanding of current technological limitations. However, this finding should be interpreted cautiously due to the limited sample size.

(2) Affordability vs. technological advancement

Families demonstrated strong sensitivity to cost and a preference for affordable devices with extended durability, likely due to financial constraints and the need for repeated device replacement during growth.

In contrast, medical professionals placed greater emphasis on technical performance and clinical applicability, including system reliability and data accessibility.

These findings suggest that a modular design approach may help balance affordability with functional complexity.

(3) Differences in service needs

Families expressed strong demand for rehabilitation guidance and training, reflecting limited knowledge of AFO-assisted rehabilitation.

Medical professionals, however, prioritized technical support and system reliability, particularly to ensure continuity of rehabilitation.

These results highlight the importance of developing integrated service models that address both educational and technical support needs.

#### 4.2.1 Influence of limited GMFCS awareness on caregiver-reported demand

A notable finding of this study was that 67.37% of families were unaware of their child’s GMFCS level. This lack of awareness may affect the interpretation of caregiver-reported functional demands. Since GMFCS provides a standardized description of gross motor function in children with cerebral palsy, limited caregiver knowledge of this classification may mean that reported needs were based mainly on daily caregiving experience rather than standardized functional assessment. As a result, some families may have overestimated or underestimated the child’s need for specific intelligent AFO functions, such as walking stability, adaptive adjustment, or data monitoring.

This knowledge gap may also introduce reporting bias. Caregivers with limited understanding of the child’s functional classification may interpret walking ability, mobility limitations, and rehabilitation needs differently from clinical professionals. Therefore, caregiver-reported demand should be interpreted as perceived practical need rather than as a direct substitute for clinically assessed functional severity. Future studies should consider confirming GMFCS levels through trained rehabilitation professionals before questionnaire completion. In addition, brief caregiver education on GMFCS and standardized functional assessment may improve the reliability and comparability of demand data. In clinical implementation, combining caregiver-reported needs with clinician-assessed functional classification may support more accurate prescription and design of intelligent AFOs.

### 4.3 Regional characteristics in Sichuan

This study highlights several regional factors that shape the demand for intelligent AFOs in underdeveloped areas of western China, emphasizing the importance of context-specific design and implementation.

(1) Economic constraints

Most families reported a monthly household income below 10,000 RMB, indicating high price sensitivity. Compared with economically developed regions, this limited affordability may restrict the adoption of high-cost intelligent AFOs. These findings underscore the importance of cost control in product development and implementation strategies.

(2) Uneven distribution of medical resources

Rehabilitation resources in Sichuan are concentrated in major urban centers, while primary healthcare institutions remain under-resourced. This imbalance may increase reliance on remote clinical support. The integration of remote monitoring and consultation functions into intelligent AFO systems may help improve accessibility and support clinical decision-making in resource-limited settings.

(3) Activity patterns and usage context

Despite the mountainous terrain of Sichuan, both families and medical professionals prioritized indoor flat-ground walking as the main application scenario for intelligent AFOs. This finding may appear inconsistent with the geographical characteristics of Sichuan; however, it should be interpreted in relation to the current functional abilities and daily activity patterns of children with cerebral palsy. For many children with CP, indoor flat-ground walking represents the most basic and frequent mobility task in daily life. Therefore, this preference may reflect current functional limitations and immediate rehabilitation priorities rather than the full range of environmental demands in Sichuan.

In other words, respondents may have prioritized the walking scenario that is most achievable, necessary, and relevant to daily caregiving, rather than more challenging outdoor or mountainous terrains. Stair climbing, slope walking, and sports participation may still be important long-term goals, but they may have been perceived as less urgent or less feasible for many children in the current sample. In addition, because the questionnaire did not explicitly frame intelligent AFOs as devices designed to support terrain adaptability, participants may not have fully considered their potential role in complex outdoor environments. Future studies should consider using scenario-based descriptions that separately assess indoor walking, stair negotiation, slope walking, uneven-ground walking, and outdoor mobility to better capture terrain-related functional demands.

### 4.4 Implications for development and implementation

Based on the identified stakeholder differences and regional characteristics, this study provides several implications for the development and implementation of intelligent AFOs.

(1) Technical considerations

Future development should prioritize lightweight design and modular functionality to enhance usability and acceptance. Core features such as fall prevention, ease of use, and adaptive adjustment should address the primary needs of families, while advanced functions such as gait monitoring and multi-joint coordination may be selectively integrated for clinical applications. This approach may help balance functionality, usability, and cost-effectiveness.

(2) Economic and policy considerations

Affordability was identified as a key determinant of demand, highlighting the need for financial support mechanisms. Policy-level interventions, such as subsidy programs or reimbursement schemes, may improve accessibility, particularly for low-income populations.

(3) Service and system integration

The findings indicate a need for integrated service models that support both families and healthcare providers. For families, structured rehabilitation guidance may improve effective device use, while for medical professionals, reliable technical support and data accessibility may enhance clinical decision-making. In resource-limited settings, remote monitoring and tele-rehabilitation may further improve service delivery.

(4) Interdisciplinary and user-centred design considerations

The development and implementation of intelligent AFOs should also be considered within a broader interdisciplinary and user-centred framework. Intelligent AFO design requires collaboration among rehabilitation physicians, physical therapists, orthotists, engineers, caregivers, and users to ensure that technological functions are aligned with real-life functional needs. From the perspective of universal design and assistive technology, both high-tech functions, such as sensor-based gait monitoring and adaptive adjustment, and low-tech design elements, such as comfort, ease of donning and doffing, durability, and affordability, should be considered together. In addition to clinical indicators, future evaluations should include user-reported outcomes, especially satisfaction, usability, perceived burden, and willingness for long-term use, because these factors directly influence acceptance and sustained use of assistive devices (10).

Overall, these implications emphasize the importance of aligning technological development with user needs, economic constraints, and healthcare system characteristics to facilitate real-world implementation.

From a broader assistive technology perspective, the design and implementation of intelligent AFOs should be informed by universal design principles and by both high-tech and low-tech assistive technology practices. Previous research on eye-tracking assistive technology for people with severe physical disabilities has highlighted the importance of user satisfaction in evaluating device usability and acceptance (10). In addition, research on adults with physical disabilities suggests that technology use may be related to motor, manual, and communication classification systems, indicating that future studies on AFOs should also consider adult users with CP and their specific functional, communication, and participation needs (11).

### 4.5 Limitations

Several limitations should be acknowledged. First, a high proportion of caregivers were unaware of their child’s GMFCS level. Although this finding reflects a real-world gap in caregiver understanding of standardized functional classification, it may have affected the reliability of caregiver-reported functional demands. The reported demand for intelligent AFO functions may have been influenced by subjective caregiving experience rather than standardized assessment of motor function, which may have introduced reporting bias and limited the ability to stratify demand according to functional severity. Future studies should incorporate clinician-confirmed GMFCS classification or standardized functional assessment before data collection.

Second, the sample size of medical professionals was relatively small, and no formal *a priori* power analysis was conducted. The inclusion of 39 medical professionals may have reduced statistical power and increased the risk of Type II error, leading to some potentially meaningful associations failing to reach statistical significance. The small sample also limited the number of independent variables that could be included in the logistic regression model. Although several candidate variables were considered, only experience with dynamic AFOs and years of clinical experience were retained in the final simplified model to reduce model complexity and avoid overfitting. As a result, omitted variable bias cannot be excluded, because other potentially relevant factors, such as professional background, monthly CP patient volume, prior AFO prescription experience, and confidence in prescription decision-making, were not included in the final model. Therefore, the simplified regression results for medical professionals should be interpreted as exploratory rather than confirmatory. The generalisability of these findings to broader groups of rehabilitation professionals or to other regions should also be considered limited. Future studies should include larger samples of medical professionals, conduct formal sample size and power calculations, and use more comprehensive regression models to verify these associations.

Third, although a standardized definition and briefing procedure were used to introduce the concept of intelligent AFOs, participants may still have varied in their understanding of this emerging technology. In particular, because no visual aids, device prototypes, or live demonstrations were provided, some participants may have interpreted functions such as adaptive adjustment, data monitoring, or fall-risk prediction differently. This variability may have influenced response validity and stakeholder expectations. Future studies should consider using standardized visual materials, device demonstrations, or scenario-based explanations to improve participants’ understanding before questionnaire completion.

Fourth, the questionnaire assessed preferred application scenarios but did not explicitly frame intelligent AFOs as terrain-adaptive devices. Therefore, the high priority given to indoor flat-ground walking may reflect current functional limitations and daily caregiving priorities rather than the absence of demand for outdoor or complex-terrain mobility. Future research should include more detailed scenario-based questions on stair climbing, slope walking, uneven-ground walking, and outdoor mobility.

Fifth, this study focused on children with CP and their families, and the findings may not fully reflect the needs of adults with CP. Adults with CP may have different functional priorities, assistive technology experiences, communication needs, and expectations related to independent living, education, employment, and social participation. More broadly, research on adults with physical disabilities suggests that technology use may be associated with motor, manual, and communication classification systems, indicating the importance of considering functional classification when evaluating assistive technology needs in adult populations (11). Future studies should examine the demand for intelligent AFOs among adults with CP and consider both quantitative indicators and qualitative user-reported outcomes, including satisfaction, usability, and long-term acceptance.

## 5 Conclusion

In conclusion, the demand for intelligent ankle-foot orthoses (AFOs) among families of children with cerebral palsy and medical professionals in Sichuan is shaped by a combination of clinical characteristics, caregiving contexts, and regional conditions.

While both groups share common priorities in safety, comfort, and scenario adaptability, clear differences exist in their emphasis on usability versus clinical functionality, affordability versus technological advancement, and service expectations. These findings highlight the importance of incorporating multi-stakeholder perspectives into the design and implementation of intelligent AFOs.

Adopting user-centered and context-specific strategies may improve both accessibility and clinical effectiveness. Furthermore, the regional insights identified in this study provide valuable evidence for the localized development and broader implementation of intelligent rehabilitation devices in resource-limited settings.

## Data Availability

The original contributions presented in the study are included in the article/Supplementary material, further inquiries can be directed to the corresponding author.
