# Supplementary material for: Determinants of demand for intelligent ankle-foot orthoses in children with cerebral palsy: a multicenter cross-sectional study from a multi-stakeholder perspective
Source: Front Public Health. 2026 Jun 9;14:1855559. doi: 10.3389/fpubh.2026.1855559 (PMC13286895; doi:10.3389/fpubh.2026.1855559)
Supplement: Supplementary file 1 [file Table_1.DOCX]

Supplementary

Supplementary Table 1. Demographic characteristics of children with cerebral palsy

| Variable | Category | *n* | Percentage（%） |
| --- | --- | --- | --- |
| Sex | Male | 49 | 51.58 |
|  | Female | 46 | 48.42 |
| Age | 0-7 years | 40 | 42.11 |
|  | 8-12 years | 28 | 29.47 |
|  | 13-17years | 18 | 18.95 |
|  | ≥18 years | 9 | 9.47 |
| GMFCS Level | Level II | 8 | 8.42 |
|  | Level IV | 9 | 9.47 |
|  | Other (Level I/III, etc.) | 14 | 14.74 |
|  | Unknown | 64 | 67.37 |
| Place of residence | Chengdu | 26 | 27.37 |
|  | Other cities/prefectures | 69 | 72.63 |
| Primary caregiver | Parents | 52 | 54.74 |
|  | Grandparents | 21 | 22.11 |
|  | Others (relatives/caregivers) | 22 | 23.15 |
| Monthly household income | <5,000 RMB | 50 | 52.63 |
|  | 5,000–10,000 RMB | 36 | 37.90 |
|  | 10,000–20,000 RMB | 9 | 9.47 |
| Living environment | Urban | 55 | 57.89 |
|  | Rural | 13 | 13.68 |
|  | Town/other | 27 | 28.43 |

Supplementary Table 2. Walking difficulties and current AFO use among children with cerebral palsy

| Variable | Category | *n* | Percentage（%） |
| --- | --- | --- | --- |
| Primary walking difficulty | Equinovarus | 25 | 26.32 |
|  | Foot drop | 12 | 12.63 |
|  | Other types | 48 | 50.53 |
|  | Other | 10 | 10.52 |
| Current use of AFO | Yes | 64 | 67.37 |
|  | No | 31 | 32.63 |
| Type of AFO | Solid AFO | 47 | 49.47 |
|  | Other types | 12 | 12.63 |
|  | Unknown | 36 | 37.90 |
| Wearing time | <2 hours/day | 47 | 49.47 |
|  | 2–8 hours/day | 34 | 35.78 |
|  | >8 hours/day | 14 | 14.75 |
| Satisfaction with AFO use | Very satisfied | 18 | 18.94 |
|  | Satisfied | 29 | 30.53 |
|  | Neutral | 36 | 37.89 |
|  | Very dissatisfied | 12 | 12.64 |

Supplementary Table 3. Price acceptability and warranty expectations for traditional AFOs

| Variable | Category | *n* | Percentage（%） |
| --- | --- | --- | --- |
| Price acceptability | <10,000 RMB | 88 | 92.63 |
|  | 10,000-30,000  RMB | 6 | 6.32 |
|  | 30,000–50,000 RMB | 1 | 1.05 |
| Warranty expectations | 1–3 years | 17 | 17.89 |
|  | 5 years | 18 | 18.95 |
|  | Lifetime | 60 | 63.16 |

Supplementary Table 4. Expectations of families regarding intelligent AFO devices

| Domain | Response | *n* | Percentage（%） |
| --- | --- | --- | --- |
| Willingness to try intelligent AFO (free trial) | Very willing | 36 | 37.89 |
|  | Willing | 40 | 42.11 |
|  | Neutral | 18 | 18.95 |
|  | Unwilling | 1 | 1.05 |
| Willingness to provide regular feedback | Very willing | 32 | 33.68 |
|  | Willing | 32 | 33.68 |
|  | Neutral | 25 | 26.32 |
|  | Unwilling | 6 | 6.32 |
| Demand for usability of intelligent AFO | Very willing | 17 | 17.89 |
|  | Willing | 31 | 32.63 |
|  | Neutral | 31 | 32.63 |
|  | Unwilling | 16 | 16.85 |
| Warranty expectations for intelligent AFO | 1–3 years | 22 | 23.16 |
|  | 5 years | 23 | 24.21 |
|  | Lifetime | 50 | 52.63 |

Supplementary Table 5. Demographic and professional characteristics of medical professionals

| Variable | Category | *n* | Percentage（%） |
| --- | --- | --- | --- |
| Sex | Female | 27 | 69.23 |
|  | Male | 12 | 30.77 |
| Age | 25-34 years | 22 | 56.41 |
|  | 35-44 years | 15 | 38.46 |
|  | ≥45 years | 2 | 5.13 |
| Place of residence | Chengdu | 16 | 41.03 |
|  | Other cities/prefectures | 23 | 58.97 |
| Professional background | Physical therapist | 18 | 46.15 |
|  | Rehabilitation physician | 15 | 38.46 |
|  | Orthotist | 3 | 7.69 |
|  | Other | 3 | 7.69 |
| Years of clinical experience | 1-4 years | 13 | 33.33 |
|  | 5-10 years | 13 | 33.33 |
|  | 11-20 years | 11 | 28.21 |
|  | ≥20 years | 2 | 5.13 |
| Monthly number of CP patients treated | ＜10 cases | 15 | 38.46 |
|  | 10-30 cases | 15 | 38.46 |
|  | 31-50 cases | 6 | 15.38 |
|  | ＞50 cases | 3 | 7.69 |
| Experience prescribing conventional AFOs | Yes | 31 | 79.49 |
|  | No | 8 | 20.51 |
| Experience with dynamic AFOs | Yes | 17 | 43.59 |
|  | No | 22 | 56.41 |
| Confidence in prescription decision-making | Very confident | 3 | 7.69 |
|  | Confident | 14 | 35.90 |
|  | Neutral | 20 | 51.28 |
|  | Not confident | 2 | 5.13 |
